# Accurate serotype identification of *Streptococcus pneumoniae* using nanopore Cas9-targeted serotype identification (nCATSerotyping)

**DOI:** 10.1128/jcm.00984-25

**Published:** 2025-12-30

**Authors:** Hyun Jung Ji, A-Yeung Jang, Seung Hyun Han, Min-Kyu Kim, Charles Euloge Lamien, Viskam Wijewardana, Ki Bum Ahn, Kyung-Hyo Kim, Joon Young Song, Ho Seong Seo

**Affiliations:** 1 Research Division for Cyclotron Application, Korea Atomic Energy Research Institute, Jeongeup, Republic of Korea; 2 Department of Oral Microbiology and Immunology, DRI, School of Dentistry, Seoul National University, Seoul, Republic of Korea; 3 Division of Infectious Disease, Department of Internal Medicine, Korea University Guro Hospital, Korea University College of Medicine, Seoul, Republic of Korea; 4 Vaccine Innovation Center-KU Medicine (VIC-K), Seoul, Republic of Korea; 5 Department of Nuclear Sciences and Applications, Animal Production and Health Laboratory, Joint FAO/IAEA Centre of Nuclear Techniques in Food and Agriculture, International Atomic Energy Agency, Seibersdorf, Austria; 6 Department of Pediatrics, Ewha Womans University Mokdong Hospital, Seoul, Republic of Korea; 7 Department of Radiation Science, University of Science and Technology, Daejeon, Republic of Korea; Endeavor Health, Evanston, Illinois, USA

**Keywords:** *Streptococcus pneumoniae*, nanopore sequencing, CRISPR-Cas9, serotyping, emerging serotypes, vaccine development

## Abstract

*Streptococcus pneumoniae* (pneumococcus) is a leading cause of community-acquired pneumonia and invasive diseases, particularly among children and the elderly. The introduction of pneumococcal conjugate vaccines has significantly reduced invasive pneumococcal disease, but the prevalence of non-vaccine serotypes and newly emerging serotypes is increasing globally. Thus, accurate serotyping is essential for epidemiological surveillance and the development of next-generation multivalent pneumococcal vaccines. Conventional serotyping methods, including multiplex polymerase chain reaction (mPCR), monoclonal antibody (mAb) assays, and Quellung reaction using rabbit antisera, are limited by serotype coverage and cross-reactivity, making the detection of new or emerging serotypes challenging. In this study, we developed a nanopore Cas9-targeted serotyping (nCATSerotyping) platform, which employs Cas9-mediated enrichment of the capsular polysaccharide synthesis locus followed by Oxford Nanopore sequencing. Applying this method to 276 clinical pneumococcal isolates collected in South Korea (2018–2020), we achieved a serotyping success rate of 97.10% (268/276), significantly outperforming conventional methods such as mAb and mPCR, which identified only 76.45% (211/276) of isolates. Whole-genome sequencing of the remaining eight non-typeable isolates revealed them to be non-pneumococcal (oral streptococci), confirming 100% accuracy for *S. pneumoniae* serotyping. Importantly, our method identified emerging and underrepresented serotypes, including serotype 13 and null capsule clade strains. nCATSerotyping offers a rapid, accurate, and comprehensive solution for pneumococcal serotyping, with significant advantages in identifying novel and non-typeable strains. This scalable platform will be a valuable tool for global serotype surveillance and next-generation multivalent pneumococcal vaccine development.

**IMPORTANCE:**

Accurate pneumococcal serotyping is critical for vaccine development and epidemiological surveillance, particularly as non-vaccine serotypes emerge following widespread pneumococcal conjugate vaccine implementation. Current serotyping methods face significant limitations in coverage and accuracy, identifying around 76% of pneumococcal isolates and failing to detect emerging serotypes like serotype 13 and null capsule clades. The nanopore Cas9-targeted serotyping platform addresses these critical gaps by achieving 100% serotyping accuracy for confirmed *Streptococcus pneumoniae* isolates while identifying previously undetectable strains that conventional methods missed. This comprehensive approach is essential for monitoring vaccine effectiveness, understanding serotype replacement patterns, and informing next-generation vaccine development strategies. Furthermore, the identification of misclassified oral streptococci highlights the diagnostic precision needed for accurate pneumococcal surveillance, ensuring that epidemiological data accurately reflect true pneumococcal disease burden and serotype distribution patterns.

## INTRODUCTION

*Streptococcus pneumoniae* (pneumococcus) is the dominant respiratory bacterial pathogen responsible for community-acquired pneumonia and invasive diseases, particularly affecting young children, older adults, and individuals with underlying medical conditions (1). The capsule covalently bound to the peptidoglycan serves as the target for current pneumococcal vaccines, which confer serotype-specific immunity (2). Although pneumococcal conjugate vaccines (PCVs) have markedly contributed to the reduction of the incidence of invasive pneumococcal diseases (IPDs), the emergence of non-vaccine serotypes (NVTs; e.g., 24F, 33F) and new serotypes (e.g., 6C, 6D, and 10D) presents significant challenges for the development of highly multivalent vaccines (e.g., PCV20, PCV21, PCV24, PCV25, and PCV31) (3–7). To date, over 107 genetically, structurally, and serologically distinct capsule types have been reported with the potential for the emergence of at least seven additional new serotypes following the introduction of highly multivalent vaccines (6, 7). Therefore, continuous surveillance is crucial to monitor vaccine efficacy, serotype replacement across various geographical regions, and to inform strategies for new vaccine development.

Several pneumococcal serotyping systems have been developed, primarily categorized into serological and genotyping assays. Serological methods like the Quellung reaction remain widely used but are limited by cross-reactivity, low specificity, and lengthy procedures (8). Despite these advances, both methods suffer from limitations such as slow processing times, low specificity, and cross-reactivity with similar serotypes. A newer approach utilizes monoclonal antibodies (mAbs) specific to the capsule, offering improved specificity and reduced cross-reactivity, which has enabled the identification of previously unrecognized serotypes (9). However, the production of mAbs for all serotypes remains challenging, with current systems covering only a limited number of serotypes. The genotyping assays have been the most widely used method (10). The multiplex polymerase chain reaction (mPCR) initially targets a small set of serotypes, with additional mPCR sets used for further identification. However, it also has the disadvantage of being difficult to find point mutations that occur in specific parts, such as 6A/B/C/D (11).

To enhance specificity and reduce processing time, our laboratory has combined mAbs detection for 27 serotypes with a four-set mPCR system covering more than 12 serotypes (Table S3). However, the potential for extensive capsule diversity post-PCV vaccination requires careful consideration of the serotyping to ensure comprehensive coverage of all known and emerging serotypes. Whole-genome sequencing (WGS) represents another genotyping approach, offering extensive data beyond serotyping, including information on MLST, antibiotic resistance, and virulence factors (12). However, traditional WGS approaches using platforms such as Illumina sequencers are expensive and time-consuming, often requiring outsourcing to specialized sequencing facilities and may occasionally fail to accurately read the capsular polysaccharide synthesis (CPS) locus. In contrast, recent advances in nanopore technology have significantly reduced sequencing costs and turnaround times, particularly when combined with targeted approaches like Cas9-mediated enrichment, making comprehensive genomic analysis more accessible for routine diagnostic applications (13). It offers long-read, direct sequencing, making it suitable for various diagnostic applications, including direct RNA sequencing and nanopore Cas9-targeted sequencing (nCATS) (14, 15). Among these, nCATS is particularly notable for achieving high sequencing depth and comprehensive variant detection within specific genomic regions. This method enriches target regions using Cas9/CRISPR to cleave chromosomal DNA, followed by sequencing of the enriched regions (16).

In this study, we successfully applied nCATS for pneumococcal serotyping. While previous serotyping methods, such as mPCR and mAb-based assays, covered 76.45% of pneumococcal isolates (*n* = 276), our nanopore Cas9-targeted serotyping (nCATSerotyping) achieved 100% coverage of these isolates. Although no new serotypes were identified in this study, the capability of our system to detect novel or variant serotypes offers advantages over conventional sequencing methods. While most newly discovered serotypes represent minor variants of established types with limited clinical impact, the identification of clinically significant variants (such as serotype 6C, initially misidentified as 6A following PCV7 implementation) demonstrates the potential value of comprehensive sequencing-based serotyping for vaccine surveillance programs.

## MATERIALS AND METHODS

### Bacterial isolates and culture conditions

All clinical isolates of *S. pneumoniae* (*n* = 276) were collected between 2018 and 2020 at 11 hospitals in South Korea. All pneumococcal isolates were cultured on blood agar plates (Kisan Bio Co., Seoul, Republic of Korea) at 37°C. For broth culture, the colonies were inoculated into Todd-Hewitt broth with 5% yeast extract (THY) and cultured at 37°C until reaching an optical density at 600 nm of 0.5. Cultures were aliquoted and stored at −80°C in THY containing 15% glycerol.

### Conventional serotyping using mAbs and mPCR

Conventional pneumococcal serotyping was performed using a Luminex-based multibead assay with serotype-specific mAbs targeting 27 pneumococcal serotypes, as described previously (17). Isolates non-typeable (NT) by the Luminex-based multibead assay underwent further analysis by mPCR targeting 14 pneumococcal serotypes (Table S3) (18). Genomic DNA was extracted for mPCR using four primer sets covering additional serotypes (Set 1: 23B, 15A, 23A; Set 2: 45, 34, 24A/F, 16F; Set 3: 29, 12F, 9N, 35B; and Set 4: 7C, 35F, 31). The *cpsA* gene was used as an internal positive control. Primers were designed based on protocols from the U.S. Centers for Disease Control and Prevention. PCR was performed under the following conditions: initial denaturation at 94°C for 4 min; 30 cycles of denaturation at 94°C for 45 s, annealing at 54°C for 45 s, and extension at 65°C for 2 min 30 s; followed by a final extension at 72°C for 5 min. Isolates not identified by either method were classified as NT.

### Spacer design for Cas9/CRISPR targeting of *dexB* and *aliA* in the CPS locus

To selectively enrich the CPS locus, two conserved flanking genes (*dexB* and *aliA*) were targeted. Spacer sequences were designed using the CHOPCHOP web tool (https://chopchop.cbu.uib.no), which generated potential guide sequences targeting the *dexB* and *aliA* regions. Four spacer sequences (dexB-Sc3001, 5′-AGT TCT ACT CTG ATC CAA AG-3′; aliA-Sc5001, 5′-AGT TTG TCC ATT CAA CTG AG-3′; dexB-Sc3002, 5′-CAA GTT CTA CTC TGA TCC AA-3′; aliA-Sc5002, 5′-TCC ATT CAA CTG AGA GGC AT-3′) were selected. Each crRNA was synthesized by linking the universal CRISPR scaffold sequence to the 3′ end of the spacer (Integrated DNA Technologies, Coralville, IA, USA).

### Genomic DNA extraction for nCATSerotyping

Genomic DNA was extracted using the G-spin Genomic DNA Extraction Kit (Intron Inc., Seoul, Republic of Korea). DNA purity was assessed by measuring the *A*_260_/*A*_280_ ratio using a spectrophotometer (Epoch 2; Biotek, Winooski, VT, USA), and DNA integrity was verified by agarose gel electrophoresis. DNA concentration was determined using the Qubit dsDNA HS Assay Kit with Qubit 4 Fluorometer (Thermo Fisher Scientific, Waltham, MA, USA). A total of 5 μg of genomic DNA was used for nCATSerotyping.

### nCATSerotyping

To assemble the Cas9 ribonucleoprotein (RNP) complex, 1 μL each of 100 μM crRNA and tracrRNA were annealed at 95°C for 5 min and cooled to room temperature. The annealed RNA duplex was incubated with 0.8 μL of HiFi Cas9 nuclease (IDT, 100 μg/μL) for 30 min at room temperature. Genomic DNA was first dephosphorylated to prevent non-specific ligation of sequencing adapters. The Cas9 RNP complex was incubated with the dephosphorylated DNA with dATP and Taq DNA polymerase at 37°C for 30 min and 72°C for 5 min to generate dA-tails. Adapters from the Ligation Sequencing gDNA-Cas9 Enrichment Kit (SQK-CS9109, Oxford Nanopore Technologies, UK) were then ligated to the target DNA. DNA fragments were purified using AMPure XP beads (Beckman Coulter, CA, USA) and eluted in 13 μL nuclease-free water (Thermo Fisher Scientific, Waltham, MA, USA). Prepared libraries were loaded onto MinION flow cells (FLO-MIN106D, R9.4.1) and sequenced using the MinION Mk1C platform.

### Nanopore WGS of NT isolates

Isolates that failed to be assigned a serotype by nCATSerotyping either due to the absence of alignment to any of the 107 known CPS locus sequences (GenBank accession: CR931632–CR931722, JF911515, HM171374, KT907353, KC832411, KC832410, KF597302, ERR051587, GU074953, SAMN14150919, JQ653094, JQ653093, PQ205320, MW683304, ERR1788088, OR509570, PQ281427, KY084476, and MK606436) or due to alignment with non-encapsulated *S. pneumoniae* (null capsule clades [NCC1–3]) (GenBank accession: JF489999 to JF490008, JF723379, and JF723380) were subjected to WGS for further characterization and species confirmation. For WGS, 1 µg of high-quality genomic DNA from each NT isolate was used to prepare sequencing libraries using the Ligation Sequencing Kit (SQK-LSK109; Oxford Nanopore Technologies) in combination with the Native Barcoding Expansion Kit (EXP-NBD104; Oxford Nanopore Technologies), allowing for multiplexing of up to 12 samples per flow cell. The DNA samples were first repaired and end-prepped following the manufacturer’s protocol, then barcoded individually using native barcodes and ligated with sequencing adapters. The barcoded libraries were pooled and purified using AMPure XP beads (Beckman Coulter), and the final library was loaded onto an R9.4.1 flow cell (FLO-MIN106D; Oxford Nanopore Technologies) for sequencing on the MinION Mk1C platform (Oxford Nanopore Technologies). The sequencing run was performed for 24 h, and raw data were basecalled in real time using Guppy (version 6.5.7) in high-accuracy mode. Post-sequencing, reads were demultiplexed and aligned against the *Streptococcus* reference genomes (GenBank accession: AE005672.3) using Minimap2. Species-level identification was carried out by comparison to a curated reference database, and isolates were classified as *S. pneumoniae*, *S. mitis*, *S. oralis*, or other oral streptococci (GenBank accession: CP135436, CP058258, CP065707, AP028929, CP152419, CP014264, CP077259, and CP097843) based on alignment coverage and sequence identity metrics.

### Bioinformatics analysis

Basecalled FASTQ reads were aligned to a custom reference FASTA file containing 107 known CPS sequences (GenBank accession: CR931632–CR931722, JF911515, HM171374, KT907353, KC832411, KC832410, KF597302, ERR051587, GU074953, SAMN14150919, JQ653094, JQ653093, PQ205320, MW683304, ERR1788088, OR509570, PQ281427, KY084476, and MK606436). Reads from unaligned isolates were then aligned against a secondary FASTA reference comprising non-encapsulated *S. pneumoniae* (NCC1–3) sequences (GenBank accession: JF489999 to JF490008, JF723379, and JF723380). Alignment was performed using the wf-alignment workflow (https://github.com/epi2me-labs/wf-alignment) on EPI2ME (Oxford Nanopore Technologies). Analysis was performed using Python 3.8.18 (https://www.python.org/) and the following tools: SeqKit v2.6.1 (19), Minimap2 v2.26-r1175 (20), Samtools v1.18 and bgzip (HTSlib v1.18) (21), Fastcat v0.15.1, Mosdepth v0.3.6 (22), Pysam v0.21.0, and Ezcharts v0.7.6. Serotype assignment was determined based on ≥90% alignment coverage and ≥95% sequence identity. For serogroups 6 and 15B/15C, specific genetic features were further analyzed. For serogroup 6 (6A, 6B, 6C, and 6D), serotype differentiation was based on the presence or absence of the *wciNβ* gene and a single nucleotide polymorphism at position 584 of the *wciP* gene. Isolates with guanine (G) at this position were classified as serotypes 6A or 6C, whereas those with adenine (A) were identified as serotypes 6B or 6D. For serogroup 15 (15B and 15C), serotype assignment was determined by analyzing the number of (TA)_*n*_ repeat units within the *wciZ* gene, downstream of the conserved sequence motif ATT TAT TTG CTA TAT T, which allowed discrimination between serotypes 15B and 15C.

## RESULTS

### The workflow of nCATSerotyping

**Fig 1:**
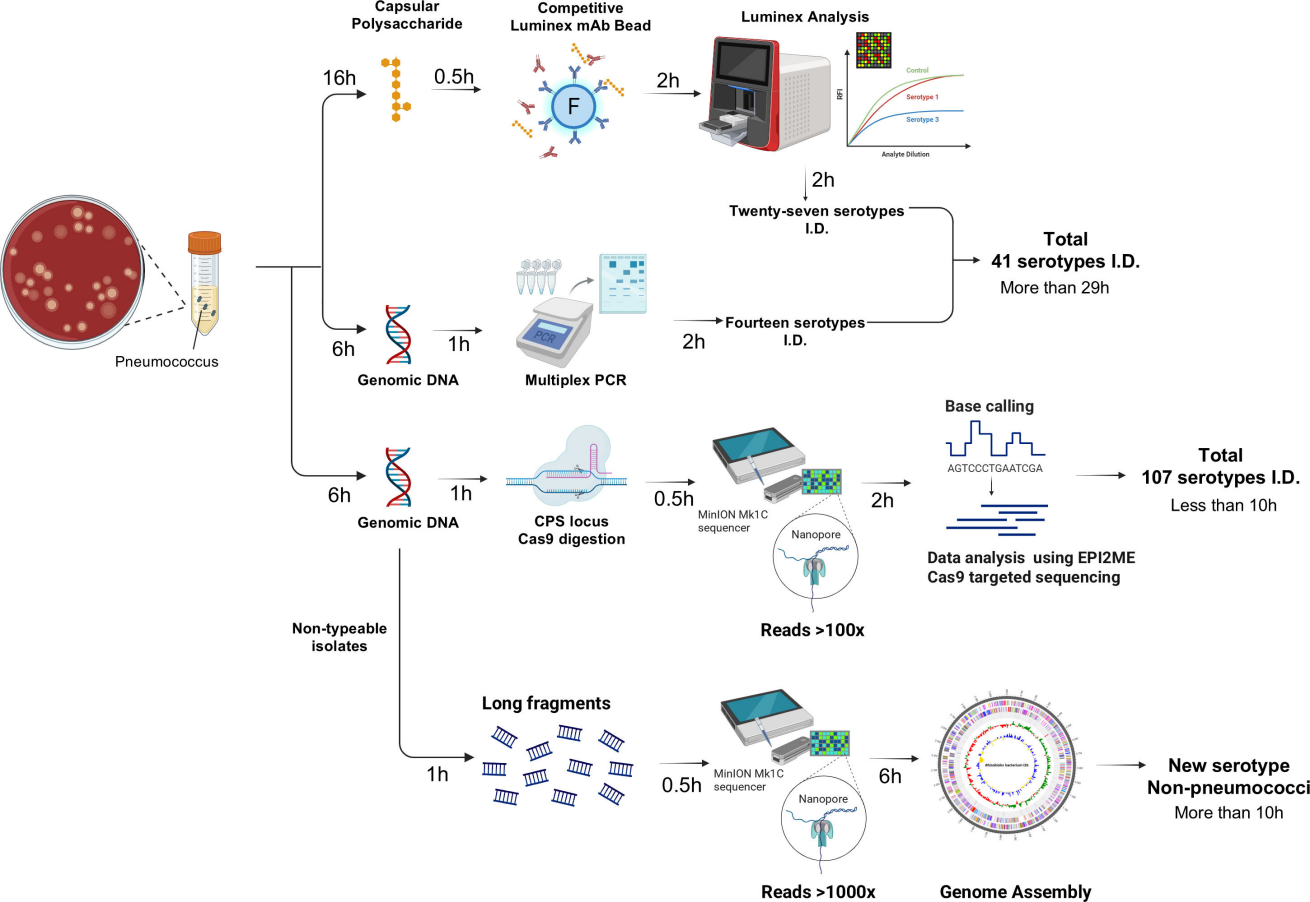
Schematic workflow for pneumococcal serotyping using conventional methods versus the nCATSerotyping platform. The upper panel illustrates traditional serotyping methods: mAb-based Luminex bead assay identifying 27 serotypes and mPCR covering an additional 14 serotypes, requiring more than 29 h. The lower panel depicts the nCATSerotyping process, which involves genomic DNA extraction, Cas9-mediated enrichment of the CPS locus, nanopore sequencing (MinION Mk1C), and bioinformatics analysis, identifying over 107 serotypes within approximately 10 h. NT isolates undergo WGS with nanopore technology, leading to the identification of new or non-pneumococcal isolates through genome assembly.

The nCATSerotyping workflow begins with genomic DNA extraction, followed by cleavage of the *dexB* and *aliA* genes using Cas9 RNP complexes to enrich the CPS locus. The enriched DNA is then sequenced using the Oxford Nanopore (Fig. 1). Resulting FAST5 files are aligned to a reference database of 107 known CPS sequences using Minimap2. In cases where there is a single nucleotide variation in the *wciP* gene (G to A at residue 584), leading to an amino acid substitution (Ser195Asn) that differentiates serotypes (6A and 6D) or (6B and 6C), or where there are variations in the number of TA repeats ([TA]_*n*_) in the *wci*Z gene for serotypes 15B and 15C, the aligned FASTA file is re-examined to confirm the serotype. A serotype 37 isolate was initially detected by analyzing the cps locus between aliA and dexB using nCATSerotyping. To avoid misclassification and ensure accuracy in serotype designation, the presence of the *tts* gene was subsequently confirmed by PCR. When nCATSerotyping failed, WGS was conducted to confirm the species. Out of 276 clinical IPD isolates collected between 2018 and 2020, nCATSerotyping successfully identified the serotype of 268 isolates (97.10%). The remaining eight isolates, unidentifiable by any method, were confirmed as oral streptococci through WGS.

### Pneumococcal clinical isolates

Between 2018 and 2020, 276 pneumococcal isolates were collected from 11 hospitals in South Korea (Table S1). By year, 2 (0.72%), 133 (48.19%), and 141 (51.09%) isolates were obtained in 2018, 2019, and 2020, respectively. The isolates were classified into three groups based on their origin: the IPD group (*n* = 60) (comprising isolates from blood, peritoneal fluid, cerebrospinal fluid, synovial fluid, and pericardial fluid), a group obtained from respiratory specimens (*n* = 199), and an “others” group (*n* = 17) for isolates sourced from all other locations (Table S2). All isolates were identified as *S. pneumoniae* following the Korea Guro hospital protocol using VITEK MS, whose sensitivity has been reported between 91.7% and 99.1% for identifying *S. pneumoniae* (23, 24).

### Comparison of classical serotyping methods with nCATSerotyping

Our laboratory implemented a pneumococcal serotyping protocol developed by Prof. Nahm at the University of Alabama at Birmingham (25). Initially, we performed serotyping using a Luminex-based method with capsule-specific mAbs. For isolates that could not be identified, we applied four sets of mPCR. Any isolates that remained unclassified after these methods were defined as NT strains. Among 276 isolates, we identified the serotypes of 134 isolates using mAbs and an additional 77 isolates using mPCR. This combined approach successfully serotyped 211 isolates, achieving a 76.45% identification rate. In contrast, when using nCATSerotyping, we successfully identified the serotypes of 268 isolates (97.10%), leaving only eight isolates unclassified (Table 1).

**TABLE 1:** Comparison of pneumococcal serotyping results obtained by three different methods: mAb-based Luminex assay, mPCR, and nCATSerotyping^*a*^^,^^*b*^

Serotypes	Monoclonal antibody	Multiplexed PCR	nCATSerotyping	# of mistyping
3	20	–	23	3
5	–	–	1	
6A	6	–	6	
6B	5	–	5	
6C	7	–	7	
6D	11	–	11	
7C		1	1	
8	1	-	1	
9N	-	2	3	1
9V	1	–	1	
10A	11	–	12	1
11A	11	–	11	
11F			0	
11E	4	–	4	
12F	–	1	1	
13	–	–	13	
14	2	–	2	
15A	–	10	10	
15B	3	–	3	
15C	–	–	3	
16F	–	3	3	
17F	3	–	3	
18C	3	–	3	
19A	20	–	20	
19F	11	–	11	
20	4	–	4	
22F	6	–	6	
23A	–	14	16	2
23B	–	6	6	
23F	5	–	6	1
24A	–	1	0	
24F			1	
28A	–	–	1	
34	–	21	22	1
35B	–	17	17	
35F	–	1	1	
36	–	–	1	
37	–	–	1	
NCC1	–		9	
NCC2	–		10	
NCC3	–		9	
NT^*c*^	65		0	
Oral streptococci	–		8	
*Total*	*276*		*276*	*9*

^*a*^ The table presents the serotype distribution among 276 clinical pneumococcal isolates collected in South Korea from 2018 to 2020. The number of isolates identified by each serotyping method and instances of misclassification (”# of mistyping”) are indicated. Oral streptococci were identified by WGS.

^*b*^ –, not determined.

^*c*^ NT, non-typeable serotypes.

To further analyze these eight unidentified isolates, we performed bacterial WGS using the nanopore platform. Surprisingly, all eight isolates that could not be serotyped by either the conventional serotyping methods (mAb and mPCR) or nCATSerotyping were identified as oral streptococci species, such as *Streptococcus mitis* and *Streptococcus oralis*. In fact, a previous study indicated that the VITEK MS misidentified oral isolates as *S. pneumoniae* (24). Thus, the nCATSerotyping method not only provided a 100% serotyping success rate but also revealed misidentified pneumococcal isolates through nanopore WGS, demonstrating its superior accuracy and reliability for pneumococcal serotyping (Table 2).

**TABLE 2:** WGS analysis of isolates unidentifiable by nCATSerotyping^*a*^

	Specimen source	Nanopore WGS		BLASTN result				
		Sequenced genome (bp)	Contig #	Homologous strain	Genome size (bp)	GenBank #	Query coverage (%)	Identity (%)
1	Respiratory (sputum)	1,972,593	7	*Streptococcus* sp. DTU_2020_1001019_1_SI_AUS_MUR_006	2,161,951	CP153436	83.64	79.30
2	Respiratory (sputum)	1,672,424	5	*Streptococcus* sp. oral taxon 061 strain F0704	1,765,730	CP058258	88.02	80.41
3	Respiratory (sputum)	1,923,430	12	*Streptococcus oralis* strain FDAARGOS_885	2,024,323	CP065707	80.78	75.80
4	IPD (blood)	1,967,004	20	*Streptococcus* sp. SN-1 (oral swab sample)	2,111,706	AP028929	71.83	67.13
5	Respiratory (sputum)	1,866,232	10	*Streptococcus* sp. SO-23-1 chromosome, complete genome	2,018,446	CP152419	83.50	78.69
6	Respiratory (sputum)	1,835,843	4	*Streptococcus* sp. oral taxon 431 strain F0610	2,177,905	CP014264	77.60	71.05
7	Respiratory (sputum)	2,082,177	7	*Streptococcus mitis* strain FDAARGOS_1456	1,868,859	CP077259	75.46	71.04
8	Respiratory (sputum)	1,914,534	1	*Streptococcus oralis* strain HP01 chromosome, complete genome	2,063,152	CP097843	85.27	80.23

^*a*^ Genomes of eight isolates were sequenced using the nanopore platform. BLASTN analyses revealed the most closely related homologous strains, their respective genome sizes (bp), GenBank accession numbers, query coverage (%), and identity (%). All isolates were identified as non-pneumococcal oral streptococci species, confirming their misidentification as *S. pneumoniae* by standard clinical diagnostic methods.

### nCATSerotyping enables improved accuracy in serotyping

To verify the accuracy of conventional serotyping methods, we compared the results of mAb assays and mPCR with those obtained by nCATSerotyping. Among the isolates that had been successfully serotyped by conventional methods, there were no discrepancies with the results from nCATSerotyping, confirming its reliability for clearly typeable strains. However, among the 65 isolates designated as NT by mAb and mPCR, nine isolates (3.26%, 9/276) appeared to have been inaccurately serotyped by the conventional assays, five by mAb assay, and four by mPCR assay. Specifically, two isolates initially reported as NT by mAb were correctly identified as serotypes 10A and 23F, and three were determined to be serotype 3. Among those classified as NT by mPCR, four were reassigned as serotypes 9N (*n* = 1), 23A (*n* = 2), and 34 (*n* = 1) (Table 1). These findings suggest that while conventional methods are generally robust within their detection range, they have inherent limitations in sensitivity. In contrast, the nCATSerotyping demonstrated 100% accuracy in serotyping, effectively overcoming issues related to sensitivity and operator variability.

**Fig 2:**
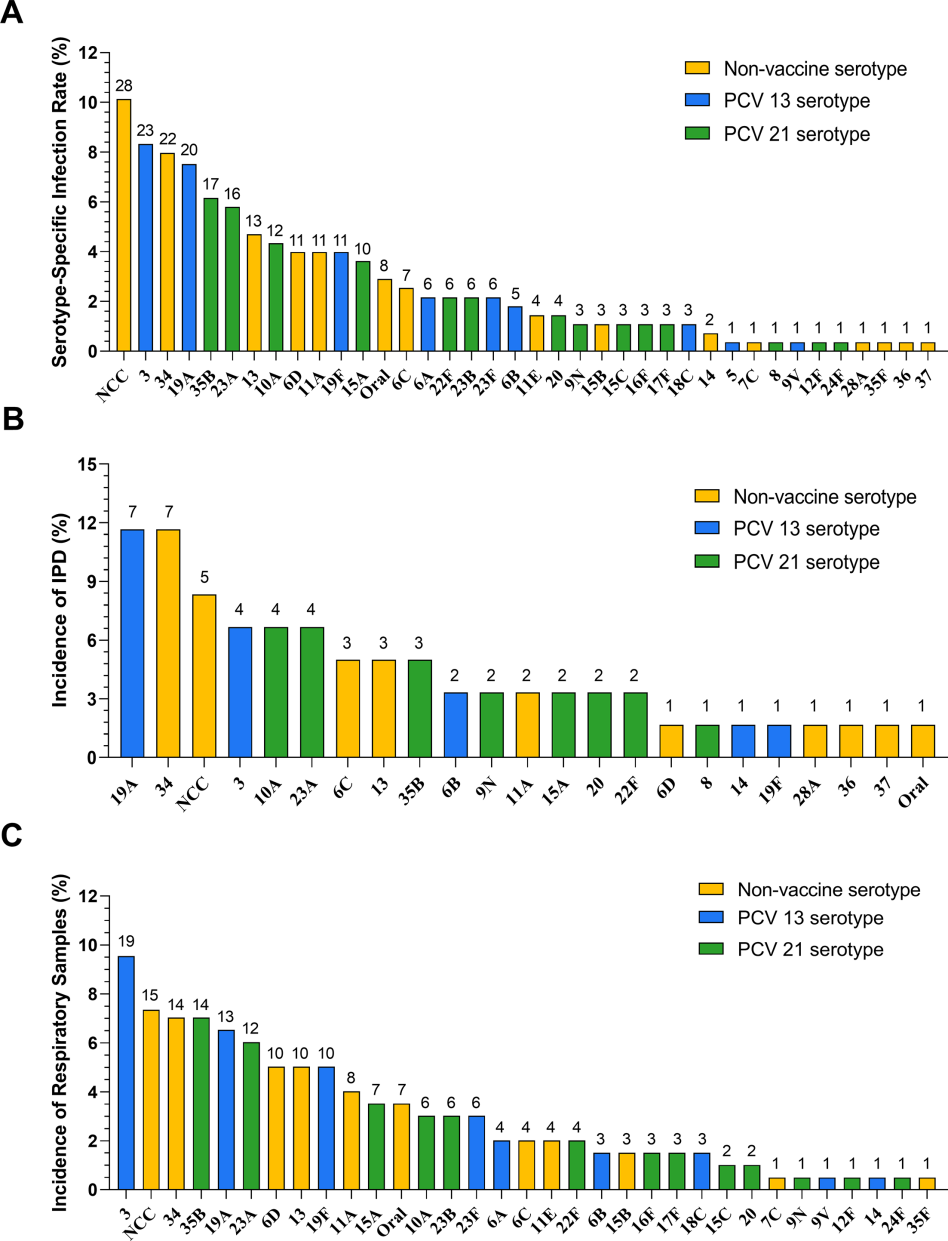
Distribution of pneumococcal serotypes identified by nCATSerotyping among clinical isolates collected in South Korea from 2018 to 2020. (**A**) Serotype distribution among all 276 clinical pneumococcal isolates. (**B**) Serotype distribution among isolates from IPD cases only (*n* = 60). (**C**) Serotype distribution among isolates obtained from respiratory specimens (*n* = 199). Serotypes are color-coded based on their inclusion in PCVs: NVTs (yellow bars), PCV13 serotypes (blue bars), and additional serotypes included in PCV21 but not in PCV13 (green bars).

Among 65 NT isolates by conventional method, 28 were identified as NCC strains (10.14%, 28/276), comprising NCC1 (*n* = 9), NCC2 (*n* = 10), and NCC3 (*n* = 9). In addition, serotype 37 (*n* = 1), serotype 36 (*n* = 1), serotype 28A (*n* = 1), serotype 15C (*n* = 3), serotype 13 (*n* = 13), and serotype 5 (*n* = 1) were assigned by nCATSerotyping. Notably, NCC (10.14%) and serotype 13 (4.71%) represented the most common serotypes among isolates that were unidentifiable by conventional methods (Table 1 and Fig. 2A). These findings highlight the utility of nCATSerotyping for accurately resolving previously undetectable or atypical pneumococcal isolates.

Among isolates obtained from cases of IPD (*n* = 60), serotype 19A and serotype 34 were identified most frequently (each 11.67%), followed by serotype 3 and serotype 23A (each 6.67%). Interestingly, NCC and serotype 13 were also detected in 8.34% and 5.00% of IPD cases (Fig. 2B). In respiratory specimens (*n* = 199), serotype 3 (9.55%), NCC (7.53%), serotype 34 (7.04%), serotype 35B (7.04%), and serotype 19A (6.53%) predominated, with serotype 13 also commonly identified (5.03%) (Fig. 2C). In contrast, nCATSerotyping of isolates from other anatomical sites besides IPD and respiratory specimens, such as bile, ear discharge, pus, conjunctiva, cornea, and vagina, indicated a much higher proportion of NCC strains (47.06%; Table 3).

**TABLE 3:** Distribution of *S. pneumoniae* serotypes by specimen source^*a*^

Serotypes	IPD		Respiratory specimen		Others	
	*n*	%	*n*	%	*n*	%
3	4	6.67	19	9.55	–	
5	–^*b*^		–		1	5.88
6A	–		4	2.01	2	11.76
6B	2	3.33	3	1.51	–	
6C	3	5.00	4	2.01	–	
6D	1	1.67	10	5.03	–	
7C	–		1	0.50	–	
8	1	1.67	–		–	
9N	2	3.33	1	0.50	–	
9V	–		1	0.50	–	
10A	4	6.67	6	3.02	2	11.76
11A	2	3.33	8	4.02	1	5.88
11F	–		–		–	
11E	–		4	2.01	–	
12F	–		1	0.50	–	
13	3	5.00	10	5.03	–	
14	1	1.67	1	0.50	–	
15A	2	3.33	7	3.52	1	5.88
15B	–		3	1.51	–	
15C	–		2	1.01	1	5.88
16F	–		3	1.01		
17F	–		3	1.51	–	
18C	–		3	1.51	–	
19A	7	11.67	13	6.53	–	
19F	1	1.67	10	5.03	–	
20	2	3.33	2	1.01	–	
22F	2	3.33	4	2.01	–	
23A	4	6.67	12	6.03	–	
23B	–		6	3.02	–	
23F	–		6	3.02	–	
24A	–		–		–	
24F	–		1	0.50	–	
28A	1	1.67	–		–	
34	7	11.67	14	7.04	1	5.88
35B	3	5.00	14	7.04	–	
35F	–		1	0.50	–	
36	1	1.67	–		–	
37	1	1.67	–		–	
NCC1	1	1.67	5	2.51	3	17.65
NCC2	1	1.67	5	2.51	4	23.53
NCC3	3	5.00	5	2.51	1	5.88
Oral streptococci	1	1.67	7	3.52	–	
*Total*	*60*	*100*	*199*	*100*	*17*	*100*

^*a*^ The number (*n*) and percentage of each pneumococcal serotype identified among clinical isolates collected from three different specimen sources: IPD, respiratory specimens, and others. Serotypes were determined using the nCATSerotyping platform.

^*b*^ –, not determined.

Another important advantage of nCATSerotyping was that some ambiguous serotypes, such as serotype 11A/F and serotype 24A/F, which cannot be distinguished by both mAb and mPCR, were serotyped correctly using nCATSerotyping. All serotype 11A/F isolates (*n* = 11) and the serotype 24A/F isolate (*n* = 1) were definitively identified as serotype 11A and serotype 24A, respectively, demonstrating that nCATSerotyping has high discriminatory capacity for distinguishing genetically and antigenically similar serotypes. Thus, our results strongly suggest that while conventional methods remain effective within their technical range, Cas9-enriched nanopore sequencing for serotype identification significantly expands the scope and precision of pneumococcal serotyping.

## DISCUSSION

*S. pneumoniae* possesses over 100 distinct capsular polysaccharide serotypes, which represent critical determinants of both virulence and immunogenicity and are the targets of current PCVs (7). The widespread implementation of PCV7 and PCV13 has significantly reduced the incidence of IPD, whereas an increasing prevalence of NVTs and newly emerging serotypes has been observed globally (26, 27). This underscores the ongoing importance of serotype surveillance in the post-vaccine era, particularly to inform the development of new, higher-valent vaccines, such as PCV20, PCV21, PCV24, PCV25, and PCV31 (5). Despite the continuous emergence of new serotypes, current serotyping tools are unable to identify the full spectrum of pneumococcal serotypes (over 107). In this study, we developed nCATSerotyping, a Cas9-based nanopore targeted-enrichment sequencing platform capable of accurate and theoretically comprehensive pneumococcal serotype deduction. By applying this method to 276 clinical pneumococcal isolates collected from 11 hospitals in South Korea between 2018 and 2020, we achieved 100% serotyping success for confirmed *S. pneumoniae* isolates. Notably, we also identified non-encapsulated pneumococci (NCC strains) and serotype 13 as emerging contributors, both of which were underdetected in conventional surveillance due to limited inclusion in routine serotyping panels (28).

nCATSerotyping offers significant advantages for novel serotype discovery through comprehensive CPS locus sequencing, although we have identified no new serotype in these clinical isolates. Our approach sequences the entire CPS locus (approximately 20 kb), enabling the detection of genetic variations that may not be captured by targeted PCR assays or conventional serological methods. For example, the discovery of serotype 6C was facilitated by similar comprehensive sequencing approaches when isolates showed negative results with traditional PCR methods but reacted with certain mAbs, leading to the identification of genetic differences in the wciN gene that distinguished 6C from 6A (29). Similarly, nCATSerotyping can identify isolates with novel genetic arrangements, insertions, deletions, or point mutations within the CPS locus that may result in new capsular structures. This capability is particularly valuable in the post-vaccine era, where selective pressure may drive the emergence of new capsular variants. Importantly, to confirm whether such genetic alterations translate into the emergence of a truly novel serotype, immunological validation through functional assays—such as opsonophagocytic killing, antibody binding, or cross-reactivity studies—will be indispensable to establish their phenotypic and clinical relevance.

A significant limitation of conventional serotyping methods lies in their incomplete serotype coverage (28, 30). Extending their range requires the development of new mAbs or additional mPCR sets, both of which increase analytical time, cost, and complexity (31). Conventional PCR-based serotyping is inexpensive and rapid but limited to predefined serotypes and often cannot resolve closely related variants (e.g., 6A/6B/6C/6D). In contrast, nCATSerotyping achieves comprehensive serotyping without the need for new reagents or workflow modifications. Moreover, while traditional serotyping methods showed a misclassification rate of approximately 3.26% (9/276) in our study, nCATSerotyping demonstrated theoretically error-free performance by directly reading the full CPS locus sequence (16). This advantage is particularly critical when distinguishing ambiguous serotypes such as 11A/F and 24A/F, which nCATSerotyping was able to resolve definitively.

Our results revealed that NCCs accounted for 10.14% (28 of 276) of pneumococcal isolates (32, 33). Despite lacking a capsule, NCCs have been increasingly associated with invasive disease, and some studies suggest the involvement of alternative virulence mechanisms (34, 35). For example, by evading the host immune response through the production of proteins that inhibit factor H binding and modulate the complement system, some NCC strains can increase the risk of IPDs in other vulnerable populations, including older adults and immunocompromised patients (36, 37). Nevertheless, the epidemiological distribution of NCCs is noteworthy because true non-encapsulated pneumococci are rare in IPD but common in carriage and non-invasive disease, with apparent NTs in IPD often representing artifacts from post-isolation capsule mutations (38). The ability of nCATSerotyping to classify these strains with high confidence provides an important tool for monitoring this emerging threat. Among the newly identified serotypes, serotype 13 was particularly notable, comprising 4.71% (13 of 276) of previously NT isolates. This serotype is not included in currently licensed PCVs and has not been widely reported in conventional surveillance data. It has recently been described in genomic studies and appears to be an emerging cause of IPD, particularly in Asia (39). Its consistent presence in our cohort underscores the need to consider serotype 13 in future vaccine formulations and surveillance efforts.

Eight isolates were NT by any method, including nCATSerotyping. Nanopore WGS revealed these to be oral streptococci species, which are frequently misidentified as *S. pneumoniae* by VITEK MS (23, 24, 40). Although oral streptococci are typically regarded as low-virulence organisms, they are increasingly implicated in invasive diseases such as infective endocarditis and IPD (41–43). In our study, only one isolate identified as *Streptococcus* sp. strain SN-1 (Table 2) was recovered from an IPD, whereas the remaining seven were isolated from respiratory specimens. Recent studies have demonstrated that some oral streptococci may produce unique capsular polysaccharides distinct from those of *S. pneumoniae*, potentially enhancing their virulence (40, 42, 44, 45). Given their possible role in invasive disease and the evolutionary convergence with pneumococcal capsule genes, future studies should investigate the genetic and structural characteristics of oral streptococcal capsules.

While nCATSerotyping demonstrated superior accuracy and reliability, the current workflow supports single-sample analysis, limiting its scalability for high-throughput surveillance. However, nanopore sequencing platforms inherently support multiplexing through barcoding, enabling the simultaneous analysis of over 96 isolates (46). Future iterations of nCATSerotyping will incorporate multiplexed library preparation to enhance throughput, reduce per-sample cost, and accelerate data acquisition. Additionally, we are extending the nCATSerotyping to other clinically important gram-positive pathogens such as *Streptococcus agalactiae* and *Streptococcus suis*, with the goal of developing a species-specific serotyping method. These efforts will further broaden the applicability of Cas9-targeted nanopore sequencing in public health and vaccine development.

One limitation of nCATSerotyping is its reliance on available cps reference sequences. Recently described variants such as serotype 6E (47), 19A/19F variants (48), and the subdivision of serogroup 20 into 20A-C were not represented in our data set. As more CPS reference sequences become available, the coverage and accuracy of this approach will continue to improve, underscoring the importance of ongoing genomic surveillance.

In summary, we present nCATSerotyping as a next-generation platform for comprehensive and highly accurate pneumococcal serotyping. This method addresses the critical limitations of current antibody-based and PCR-based approaches, enabling precise identification of both known and emerging serotypes, including NCC strains and serotype 13. Furthermore, it facilitates the detection of misidentified isolates and offers potential for broader applications across other bacterial species. With future enhancements for multiplexed analysis, nCATSerotyping holds significant promise for global serotype surveillance and next-generation vaccine design.

## SUPPLEMENTAL MATERIAL

Tables S1 to S3 jcm.00984-25-s0001.docx Table S1: Pneumococcal clinical isolates collected from 11 Korean hospitals between 2018 and 2020. A total of 276 clinical isolates were obtained from patients at 11 hospitals in South Korea over a three-year period. Table S2: Yearly distribution and specimen source classification of clinical *Streptococcus pneumoniae* isolates collected between 2018 and 2020. A total of 276 clinical isolates were obtained from patients at 11 hospitals in South Korea over a three-year period. Isolates were categorized by specimen source: invasive pneumococcal disease (IPD), respiratory specimens, and others. Table S3: Serotype coverage using mAb and mPCR set.

ASM does not own the copyrights to Supplemental Material that may be linked to, or accessed through, an article. The authors have granted ASM a non-exclusive, world-wide license to publish the Supplemental Material files. Please contact the corresponding author directly for reuse.
