# Supplementary material for: Accurate serotype identification of Streptococcus pneumoniae using nanopore Cas9-targeted serotype identification (nCATSerotyping)
Source: J Clin Microbiol. 2025 Dec 30;64(2):e00984-25. doi: 10.1128/jcm.00984-25 (PMC12892971; doi:10.1128/jcm.00984-25)
Supplement: Tables S1 to S3 — Table S1: Pneumococcal clinical isolates collected from 11 Korean hospitals between 2018 and 2020. A total of 276 clinical isolates were obtained from patients at 11 hospitals in South Korea over a three-year period. Table S2: Yearly distribution and specimen source classification of clinical Streptococcus pneumoniae isolates collected between 2018 and 2020. A total of 276 clinical isolates were obtained from patients at 11 hospitals in South Korea over a three-year period. Isolates were categorized by specimen source: invasive pneumococcal disease (IPD), respiratory specimens, and others. Table S3: Serotype coverage using mAb and mPCR set. [file jcm.00984-25-s0001.docx]

**Supplement Table 1. Pneumococcal clinical isolates collected from 11 Korean hospitals between 2018 and 2020.** A total of 276 clinical isolates were obtained from patients at 11 hospitals in South Korea over a three-year period.

| **Specimen source** | **2018** | **2019** | **2020** | **Total** |
| --- | --- | --- | --- | --- |
| Korea University Guro Hospital | 0 | 110 | 44 | 154 |
| Korea University Anam Hospital | 0 | 10 | 58 | 68 |
| Korea University Ansan Hospital | 1 | 5 | 13 | 19 |
| Hallym University Gangnam Hospital | 1 | 3 | 9 | 13 |
| Inha University Hospital | 0 | 1 | 4 | 5 |
| Chungbuk National University Hospital | 0 | 1 | 4 | 5 |
| Wonju Severance Christian Hospital | 0 | 0 | 4 | 4 |
| Jeju Halla Hospital | 0 | 1 | 3 | 4 |
| Konyang University Hospital | 0 | 1 | 1 | 2 |
| Dong-A University Hospital | 0 | 0 | 1 | 1 |
| Hallym University Dongtan Hospital | 0 | 1 | 0 | 1 |
| **Total** | **2** | **133** | **141** | **276** |

**Supplement Table 2. Yearly distribution and specimen source classification of clinical *Streptococcus pneumoniae* isolates collected between 2018 and 2020.** A total of 276 clinical isolates were obtained from patients at 11 hospitals in South Korea over a three-year period. Isolates were categorized by specimen source: invasive pneumococcal disease (IPD), respiratory specimens, and others.

| **Specimen source** | **2018** | **2019** | **2020** | **Total** |
| --- | --- | --- | --- | --- |
| **IPD** | 0  (0%) | 16  (5.80%) | 44  (15.94%) | **60**  **(21.74%)** |
| **Respiratory specimen** | 2  (0.72%) | 105  (38.04%) | 92  (33.33%) | **199**  **(72.10%)** |
| **Others** | 0  (0%) | 12  (4.35%) | 5  (1.81%) | **17**  **(6.16%)** |
| **Total** | **2**  **(0.72%)** | **133**  **(48.19%)** | **141**  **(51.09%)** | **276**  **(100%)** |

**Supplement Table 3. Serotype coverage using mAb and mPCR set**

| **Method** | **Serotypes** | |
| --- | --- | --- |
| **Monoclonal**  **Antibody** | 1, 2, 3, 4, 5, 6A, 6B, 6C, 6D, 7F, 8, 9N, 9V, 10A, 11A,  11E, 12F, 14, 15B, 17F, 18C, 19A, 19F, 20, 22F, 23F, 33F | |
| **Multiplexed**  **PCR** | Set1 | 23B (216 bp), 15A (436 bp), 23A (722 bp), *cpsA* (160 bp) |
|  | Set2 | 45 (238 bp), 34 (408 bp), 24A/F (686 bp), 16F (988 bp), *cpsA* (160 bp) |
|  | Set3 | 29 (259 bp), 12F (376 bp), 9N (516 bp), 35B (677 bp), *cpsA* (160 bp) |
|  | Set4 | 7C (260 bp), 35F (517 bp), 31 (701 bp), *cpsA* (160 bp) |
